# Supplementary material for: Evolution of Philodendron (Araceae) species in Neotropical biomes
Source: PeerJ. 2016 Mar 24;4:e1744. doi: 10.7717/peerj.1744 (PMC4811177; doi:10.7717/peerj.1744)
Supplement: Supplemental Information 1 [file peerj-04-1744-s001.docx]

**Supplementary Material**

**Table 1**. List of species, voucher information, biome and continental distribution. The species with no associated voucher corresponds to the ones that the sequences were already available in GenBank.

| **Species** | **Voucher - Herbarium** | **Biome** | **Continent** |
| --- | --- | --- | --- |
| **Outgroup** |  |  |  |
| *Cercestis* *afzelii* Schott | Chase 11690 - RBG Kew | African Tropical Moist Forest | Africa |
| *Cercestis camerunense* (Ntépé-Nyamè) Bogner | Salazar 6305 - RBG Kew | African Tropical Moist Forest | Africa |
| *Cercestis kawennianus* (Engl.) N.E. Br. | Salazar 6306 - RBG Kew | African Tropical Moist Forest | Africa |
| *Cercestis* sp. | Morais 57 | African Tropical Moist Forest | Africa |
| *Culcasia* *rotundifolia* Bogner | Morais 58 | African Tropical Moist Forest | Africa |
| *Dieffenbachia* *elegans* A.M.E. Jonker & Jonker | Calazans 88 - RFA | Amazon Forest | South America |
| *Furtadoa* *mixta* (Ridl.) M. Hotta | Sakuragui 1402 - RBG Kew | Asian Tropical Moist Forest | Asia |
| *Heteropsis* *flexuosa* (Kunth) G.S. Bunting | Morais 81 - RFA | Atlantic Forest Amazon Forest | South America |
| *Montrichardia* *arborescens* (L.) Schott | Oliveira 53 - RFA | Atlantic Forest Amazon Forest Cerrado | South America |
| *Nephthytis* *afzelii* Schott | Chase 10692 | African Tropical Moist Forest | Africa |
| *Nephthytis* *poissoni* (Engl.) N.E.Br. | Chase 14862 - RBG Kew | African Tropical Moist Forest | Africa |
| *Nephthytis* *swainei* Bogner | Chase 14863 - RBG Kew | African Tropical Moist Forest | Africa |
| *Urospatha* *sagittifolia* (Rudge) Schott | Oliveira 47 - RFA | Amazon Forest Cerrado Caatinga | South America |
| ***Homalomena*** |  |  |  |
| *H*. *aromatica* (Spreng.) Schott | Sakuragui 1368 - RBG Kew | Asian Tropical Moist Forest | South America |
| *H.* *cochinchinensis* Engl. | Calazans 36 et al. - RB; Sakuragui 1369 - RBG Kew | Asian Tropical Moist Forest | Asia |
| *H. crinipes* Engl. |  | Amazon Forest | South America |
| *H. erythropus* (Mart. ex Schott) Engl. | - | Amazon Forest | South America |
| *H. expedita* A.Hay & Hersc. | - | Asian Tropical Moist Forest | Asia |
| *H. griffithii* (Schott) Hook. f. | - | Asian Tropical Moist Forest | Asia |
| *H. humilis* (Jack) Hook. f. | Sakuragui 1371 - RBG Kew | Asian Tropical Moist Forest | Asia |
| *H. magna* A. Hay | Chase 10691 - RBG Kew | Asian Tropical Moist Forest | South America |
| *H. panamense* K. Krause | - | Unknown | South America |
| *H. pendula* (Blume) Bakh. f. | Sakuragui 1372 - RBG Kew | Asian Tropical Moist Forest | Asia |
| *H. philippinensis* Engl. | - | Asian Tropical Moist Forest | Asia |
| *H. picturata* (Linden & André) Regel | - | Amazon Forest | South America |
| *H. rubescens* (Roxb.) Kunth |  | Asian Tropical Moist Forest | Asia |
| *H. tenuispadix* Engl. | Sakuragui 1404 - RBG Kew | Asian Tropical Moist Forest | Asia |
| *H. wallichii* Schott | Sakuragui 1405 - RBG Kew | Asian Tropical Moist Forest | Asia |
| *H. wendlandii* Schott | - | Amazon Forest | Asia |
| ***P*. subg. *Meconostigma*** |  |  |  |
| *P. adamantinum* Mart. ex Schott | Bastos 20 et al., Calazans 40 - RFA | Cerrado | South America |
| *P. bipinnatifidum* Schott ex Endl. | Calazans 4 - RB | Atlantic Forest Cerrado | South America |
| *P. brasiliense* Engl. | Bastos 21 et al., Calazans 23 - RFA | Cerrado | South America |
| *P. corcovadense* Kunth | Calazans 17 et al., Morais 23 - RFA | Atlantic Forest | South America |
| *P. dardanianum* Mayo | Calazans 22 et al. - RB | Cerrado | South America |
| *P. goeldii* G.M. Barroso | Calazans 72 et al. - RB | Amazon Forest | South America |
| *P. leal-costae* Mayo & G.M. Barroso | Calazans 47 - HUEFS | Atlantic Forest Caatinga | South America |
| *P. lundii* Warm. | Calazans 41 et al. - RB | Cerrado | South America |
| *P. mello-barretoanum* R. Burle-Marx ex G.M. Barroso | Morais 51 - RB | Cerrado | South America |
| *P. paludicola* E.G. Gonç. & Salviani | Calazans 38 et al. - RB | Atlantic Forest | South America |
| *P. petraeum* Chodat & Vischer | Calazans 28 et al. - RB | Cerrado | South America |
| *P. saxicola* K. Krause | Calazans 50 & Morais - RFA | Cerrado | South America |
| *P. solimoesense* A.C. Sm. | Oliveira et al. 57 - INPA | Amazon Forest | South America |
| *P. speciosum* Schott ex Endl. | Morais 38 - RFA | Atlantic Forest | South America |
| *P. stenolobum* E.G. Gonç. | Bastos 22 et al. - RFA | Atlantic Forest | South America |
| *P. tweedieanum* Schott | Calazans 37 et al. - RB | Cerrado | South America |
| *P. uliginosum* Mayo | Calazans 19 et al. - RB | Cerrado | South America |
| *P. undulatum* Engl. | Calazans 7 - RB | Atlantic Forest Cerrado | South America |
| *P. venezuelense* G.S. Bunting | Calazans 26 - RB | Amazon Forest | South America |
| *P. williamsii* Hook. f. | Calazans 54 & Morais - HUEFS | Atlantic Forest | South America |
| *P. xanadu* Croat, Mayo & J. Boos | L. Mayano s/n | Unknown | Unknown |
| ***P*. subg. *Philodendron*** |  |  |  |
| *P. acutatum* Schott | Bastos 14, Calazans 10 - RB | Atlantic Forest Amazon Forest Cerrado Caatinga | South America |
| *P. aemulum* Schott | Sakuragui 629 - RBG Kew | Atlantic Forest | South America |
| *P. angustilobum* Croat & Grayum | Sakuragui 641 - RBG Kew | Amazon Forest | Central America |
| *P. angustisectum* Engl. | - | Amazon Forest | South America |
| *P. annulatum* Croat | Sakuragui 1378 - RBG Kew | Amazon Forest | Central America |
| *P. appendiculatum* Nadruz & Mayo | Calazans 105, Calazans 133 - RB | Atlantic Forest | South America |
| *P. asplundii* Croat & M.L. Soares | Morais 91 - RB | Amazon Forest | South America |
| *P. auriculatum* Standl. & L.O. Williams | Sakuragui 1379 - RBG Kew | Amazon Forest Cerrado | South America |
| *P. barrosoanum* G.S. Bunting | Oliveira 41 - RB | Amazon Forest | South America |
| *P. billietiae* Croat | Oliveira 38, Oliveira 40 - RB | Amazon Forest | South America |
| *P. brevispathum* Schott | - | Amazon Forest Cerrado | South America and Central America |
| *P. burlemarxii* G.M. Barroso | Bastos 5 - RB | Amazon Forest | South America |
| *P. callosum* K. Krause | Bastos 12 - RB; Sakuragui 632 - RBG Kew | Amazon Forest | South America |
| *P. camposportanum* G.M. Barroso | Bastos 23 - RB | Amazon Forest Cerrado | South America |
| *P. cannifolium* (Dryand. ex Sims) Sweet | - | Amazon Forest | South America |
| *P. cordatum* Kunth | Calazans 59, Wängler 268 | Atlantic Forest | South America |
| *P. crassinervium* Lindl. | Calazans 13 - RB; Sakuragui 1383 - RBG Kew | Atlantic Forest | South America |
| *P. davidsonii* Croat | - | Amazon Forest | Central America |
| *P. deltoideum* Poepp. | Chase 10891 - RBG Kew | Amazon Forest | South America |
| *P. distantilobum* K. Krause | - | Amazon Forest | South America |
| *P. edmundoi* G.M. Barroso | Calazans 30, Calazans 43 - RB | Atlantic Forest | South America |
| *P. elaphoglossoides* Schott | Morais 105 - RB | Amazon Forest | South America and Central America |
| *P. erubescens* K. Koch & Augustin | Pellegrini 10 - RB | Amazon Forest | South America |
| *P. eximium* Schott | Pellegrini 109, Calazans 126 - RB | Atlantic Forest | South America |
| *P. findens* Croat & Grayum | - | Amazon Forest | South America and Central America |
| *P. fragrantissimum* (Hook.) G. Don | Morais 64 - RB; Sakuragui 1385 - RBG Kew | Atlantic Forest Amazon Forest | South America |
| *P. glaziovii* Hook. f. | Sakuragui 639 - RBG Kew | Atlantic Forest | South America |
| *P. gloriosum* André | - | Amazon Forest | South America |
| *P. grandifolium* (Jacq.) Schott | Bastos 16 - RB | Amazon Forest | South America/ Andes |
| *P. grandipes* K. Krause | - | Amazon Forest Cerrado | South America and Central America |
| *P. hastatum* K. Koch & Sellow | Pellegrini 57 - RB | Atlantic Forest | South America |
| *P. hederaceum* (Jacq.) Schott | Calazans 154 - RB | Atlantic Forest Amazon Forest | South America and Central America |
| *P. heleniae* Croat | - | Amazon Forest | South America and Central America |
| *P. hopkinsianum* M.L. Soares & Mayo | Oliveira 34, Morais 106 - RB | Amazon Forest | South America |
| *P. hylaeae* G.S. Bunting | Oliveira 30 - RB | Amazon Forest | South America |
| *P. imbe* Schott ex Endl. | - | Atlantic Forest | South America |
| *P. inconcinnum* Schott | Sakuragui 636 - RBG Kew | Amazon Forest | South America |
| *P. insigne* Schott | Calazans 29 - RB; Sakuragui 1389 - RBG Kew | Atlantic Forest Amazon Forest | South America |
| *P. krugii* Engl. | Sakuragui 638 - RBG Kew | Amazon Forest | Central America |
| *P. lazorii* Croat | - | Amazon Forest | Central America |
| *P. lindenii* Schott | - | Amazon Forest | South America |
| *P. loefgrenii* Engl. | Bastos 28 - RB | Atlantic Forest | South America |
| *P. longilaminatum* Schott | Calazans 25 - RB; Sakuragui 1392 - RBG Kew | Atlantic Forest | South America |
| *P. longistilum* K. Krause | Bastos 10 - RB | Amazon Forest | South America |
| *P. malesevichiae* Croat | - | Amazon Forest | South America and Central America |
| *P. martianum* Engl. | Calazans 32 - RB | Atlantic Forest | South America |
| *P. maximum* K. Krause | Calazans 77 - RB | Amazon Forest | South America |
| *P. megalophyllum* Schott | Oliveira 44, Morais 89 - RB | Amazon Forest Cerrado | South America |
| *P. melanochrysum* Linden & André | Sakuragui 1393 - RBG Kew | Amazon Forest | South America |
| *P. melinonii* Brongn. ex Regel | Calazans 33, Morais 88 - RB | Amazon Forest | South America |
| *P. micranthum* Poepp. ex Schott | Bastos 27 - RB | Amazon Forest | South America |
| *P. myrmecophyllum* Engl. | Sakuragui 640 - RBG Kew | Amazon Forest | South America |
| *P. nadruzianum* Sakur. | Calazans 58, Calazans 62 - RB | Atlantic Forest | South America |
| *P. ornatum* Schott | Oliveira 51, Calazans 103 - RB | Atlantic Forest Amazon Forest Caatinga | South America and Central America |
| *P. pachyphyllum* K. Krause | Calazans 53 - RB | Cerrado | South America |
| *P. panamense* K. Krause | - | Amazon Forest | Central America |
| *P. panduriforme* (Kunth) Kunth | Morais 69 - RB | Amazon Forest | South America/ Andes |
| *P. pedatum* (Hook.) Kunth | Calazans 48, Morais 82 - RB | Atlantic Forest Amazon Forest Cerrado Caatinga | South America |
| *P. pinnatifidum* (Willd.) Schott | - | Amazon Forest Cerrado | South America |
| *P. pulchrum* G.M. Barroso | Morais 93 - RB | Amazon Forest | South America |
| *P. quinquelobum* K. Krause | Morais 83, Calazans 74 - RFA | Amazon Forest | South America |
| *P. radiatum* Schott | - | Amazon Forest | South America and Central America |
| *P. recurvifolium* Schott | Sakuragui 1003 - RBG Kew | Atlantic Forest | South America |
| *P. renauxii* Reitz | - | Atlantic Forest | South America |
| *P. rhizomatosum* Sakur. & Mayo | Sakuragui 637 - RBG Kew | Cerrado | South America |
| *P. roseopetiolatum* Nadruz & Mayo | Calazans 106 - RFA | Atlantic Forest | South America |
| *P. ruizii* Schott | - | Amazon Forest | South America |
| *P. ruthianum* Nadruz | Antas 10 - RB | Atlantic Forest | South America |
| *P. sagittifolium* Liebm. | - | Amazon Forest | South America and Central America |
| *P. simmondsii* Mayo | Sakuragui 642 - RBG Kew | Amazon Forest | South America and Central America |
| *P. smithii* Engl. | - | Amazon Forest | Central America |
| *P. sphalerum* Schott | Calazans 76, Calazans 95 - RFA | Amazon Forest | South America |
| *P. squamiferum* Poepp. | Morais 63 - RFA; Sakuragui 1400 - RBG Kew | Amazon Forest | South America |
| *P. stenophyllum* K. Krause | - | Amazon Forest | South America |
| *P. tortum* M.L. Soares & Mayo | Oliveira 36 - RFA | Amazon Forest | South America |
| *P. toshibai* M.L. Soares & Mayo | Oliveira 49 - RFA | Amazon Forest | South America |
| *P. tripartitum* (Jacq.) Schott | Chase 10894 - RBG Kew | Amazon Forest | South America and Central America |
| *P. verrucosum* L. Mathieu ex Schott | - | Amazon Forest | South America and Central America |
| *P. wendlandii* Schott | - | Amazon Forest | South America and Central America |
| *P. wittianum* Engl. | Oliveira 32 - RB | Amazon Forest | South America |
| *P. wurdackii* G.S. Bunting | Morais 54 - RFA | Amazon Forest | South America |
| *P. linnaei*Kunth, Enum | Calazans 27, Bastos 17 - RB | Amazon Forest Cerrado | South America and Central America |
| ***P*. subg. *Pteromischum*** |  |  |  |
| *P. oblongum* (Vell.) Kunth | Morais 48, Calazans 127 - RFA | Atlantic Forest Amazon Forest Cerrado | South America |
| *P. ochrostemon* Schott | Calazans 119 - RFA | Atlantic Forest | South America |
| *P. placidum* Schott |  | Amazon Forest | South America |
| *P. propinquum* Schott | Calazans 122 - RFA | Atlantic Forest | South America |
| *P. rudgeanum* Schott | Calazans 156, Calazans 157 - RFA | Atlantic Forest Amazon Forest | South America |
| *P. schottii* subsp. *talamancae* (Engl.) Grayum | - | Amazon Forest | South America and Central America |
| *P. surinamense* (Miq.) Engl. | Sakuragui 1001 - RBG Kew | Atlantic Forest Amazon Forest | South America |

**Table 2**. Taxon sampling and GenBank accession numbers of *Philodendron*, *Homalomena* and outgroup species.


| **Taxon sampling** | **5’ *mat*K** | **3’ *mat*K and *trn*K intron** | ***trn*L intron** | ***trn*L-*trn*F intergenic spacer** | **ETS + 18S** |
| --- | --- | --- | --- | --- | --- |
| **Outgroup** |  |  |  |  |  |
| *Cercestis* *afzelii* Schott | - | - | KU727619 | KU727678 | - |
| *Cercestis camerunensis* (Ntépé-Nyamè) Bogner | - | - | KU727597 | KU727677 | - |
| *Cercestis kawennianus* (Engl.) N.E. Br. | - | - | KU727616 | KU727675 | - |
| *Cercestis* sp. | KU739335 | - | KU727617 | KU727676 | - |
| *Culcasia* *rotundifolia* Bogner | - | - | KU727618 | KU727682 | - |
| *Dieffenbachia* *elegans* A.M.E. Jonker & Jonker | KU739301 | KU739349 | - | KU727681 | - |
| *Furtadoa* *mixta* (Ridl.) M. Hotta | KU739329 | - | KU727594 | KU727627 | - |
| *Heteropsis* *flexuosa* (Kunth) G.S. Bunting | KU739303 | - | KU727527 | KU727579 | - |
| *Montrichardia* *arborescens* (L.) Schott | KU739300 | - | KU727526 | KU727576 | - |
| *Nephthytis* *afzelii* Schott | KU739336 | KU739351 | KU727599 | KU727674 | - |
| *Nephthytis* *poissoni* (Engl.) N.E.Br. | KU739334 | - | KU727622 | KU727680 | - |
| *Nephthytis* *swainei* Bogner | - | - | KU727623 | KU727679 | - |
| *Urospatha* *sagittifolia* (Rudge) Schott | - | KU739317 | - | KU727577 | - |
| ***Homalomena*** |  |  |  |  |  |
| *H*. *aromatica* (Spreng.) Schott | - | - | KU727624 | KU727669 | - |
| *H.* *cochinchinensis* Engl. | KF971331 | KF981856 | KU727591 | KU727657 | DQ870560.1 |
| *H. crinipes* Engl. | - | - | - | - | DQ870561.1 |
| *H. erythropus* subsp. *allenii* (Mart. ex Schott) Engl. | - | - | - | - | DQ870562.1 |
| *H. expedita* A.Hay & Hersc. | JX024965.1 | JX024965.1 | - | KU727631 | - |
| *H. griffithii* (Schott) Hook. f. | KU739327 | - | - | - | - |
| *H. humilis* (Jack) Hook. f. | KU739328 | - | KU727592 | KU727659 | - |
| *H. magna* A. Hay | AM920596.1 | AM920596.1 | KU727614 | KU727683 | - |
| *H. panamensis* K. Krause | - | - | - | - | DQ870563.1 |
| *H. pendula* (Blume) Bakh. f. | - | - | KU727593 | KU727660 | - |
| *H. philippinensis* Engl. | - | - | - | - | DQ870564.1 |
| *H. picturata* (Linden & André) Regel | - | - | - | - | DQ870565.1 |
| *H. rubescens* (Roxb.) Kunth | KU739290 | - | KU727520 | KU727547 | DQ870566.1 |
| *H. tenuispadix* Engl. | KU739326 | - | KU727598 | KU727653 | - |
| *H. wallichii* Schott | KU739324 | - | KU727625 | KU727645 | - |
| *H. wendlandii* Schott | KU739283 | - | KU727528 | KU727543 | DQ870567.1 |
| ***P*. subg. *Meconostigma*** |  |  |  |  |  |
| *P. adamantinum* Mart. ex Schott | KU739331 | - | KU727607 | KU727629 | KF895425 |
| *P. bipinnatifidum* Schott ex Endl. | KF971323 | KF981849 | KU727609 | KU727567 | KF895410 |
| *P. brasiliense* Engl. | KU739325 | - | KU727513 | KU727551 | KF895413 |
| *P. corcovadense* Kunth | KF971324 | KF981850 | KU727515 | KU727568 | KF895417 |
| *P. dardanianum* Mayo | - | - | KU727587 | KU727661 | KF895411 |
| *P. goeldii* G.M. Barroso | - | - | - | KU727662 | KF895428 |
| *P. leal-costae* Mayo & G.M. Barroso | - | - | - | KU727630 | KF895427.1 |
| *P. lundii* Warm. | KF971332 | KF971332 | KU727512 | KU727546 | KF895420.1 |
| *P. mello-barretoanum* R. Burle-Marx ex G.M. Barroso | - | KU739347 | KU727588 | KU727655 | KF895423 |
| *P. paludicola* E.G. Gonç. & Salviani | - | - | KU727612 | KU727654 | KF895412 |
| *P. petraeum* Chodat & Vischer | KF981853 | KF981853 | - | KU727667 | KF895422 |
| *P. saxicola* K. Krause | KF971327 | KF981854 | KU727521 | KU727545 | KF895426 |
| *P. solimoesense* A.C. Sm. | KF971333 | KF971333 | KU727621 | KU727668 | KF895429 |
| *P. speciosum* Schott ex Endl. | - | - | KU727590 | KU727684 | KF895414 |
| *P. stenolobum* E.G. Gonç. | KF971334 | KF971334 | KU727608 | KU727664 | KF895424 |
| *P. tweedieanum* Schott | - | - | KU727611 | KU727656 | KF895421 |
| *P. uliginosum* Mayo | - | - | - | KU727651 | KF895419 |
| *P. undulatum* Engl. | KF971328 | KF981855 | KU727508 | KU727663 | KF895418 |
| *P. venezuelense* G.S. Bunting | KF971329 | KF971329 | KU727610 | KU727665 | KF895415 |
| *P. williamsii* Hook. f. | KF971330 | KF971330 | KU727589 | KU727666 | KF895416 |
| *P. xanadu* Croat, Mayo & J. Boos | - | - | KU727613 | KU727649 | KF895409 |
| ***P*. subg. *Philodendron*** |  |  |  |  |  |
| *P. acutatum* Schott | KU739289 | KU739342 | KU727510 | KU727549 | DQ870570.1 |
| *P. aemulum* Schott | KU739338 | - | - | KU727641 | - |
| *P. angustilobum* Croat & Grayum | - | - | KU727583 | KU727634 | - |
| *P. angustisectum* Engl. | - | - | - | - | DQ870576.1 |
| *P. annulatum* Croat | KU739337 | KU739350 | KU727580 | KU727647 | - |
| *P. appendiculatum* Nadruz & Mayo | KU739332 | KU739313 | KU727503 | KU727539 | - |
| *P. asplundii* Croat & M.L. Soares | KU739296 | - | KU727497 | KU727537 | - |
| *P. auriculatum* Standl. & L.O. Williams | KU739322 | - | KU727595 | KU727648 | - |
| *P. barrosoanum* G.S. Bunting | KU739282 | - | - | KU727558 | DQ870577.1 |
| *P. billietiae* Croat | KU739298 | KU739309 | KU727509 | KU727531 | DQ870578.1 |
| *P. brevispathum* Schott | - | - | - | - | DQ870579.1 |
| *P. burlemarxii* G.M. Barroso | - | KU739306 | KU727499 | KU727560 | - |
| *P. callosum* K. Krause | KU739285 | - | KU727518 | KU727673 | DQ870580.1 |
| *P. camposportanum* G.M. Barroso | - | - | - | KU727571 | - |
| *P. cannifolium* (Dryand. ex Sims) Sweet | - | - | - | - | DQ870581.1 |
| *P. cordatum* Kunth | KU739277 | KU739304 | KU727487 | KU727540 | - |
| *P. crassinervium* Lindl. | KU739330 | KU739346 | KU727596 | KU727670 | DQ870582.1 |
| *P. davidsonii* Croat | - | - | - | - | DQ870583.1 |
| *P. deltoideum* Poepp. | AM920597.1 | AM920597.1 | KU727585 | KU727646 | - |
| *P. distantilobum* K. Krause | - | - | - | - | DQ870584.1 |
| *P. edmundoi* G.M. Barroso | KU739295 | - | - | KU727570 | - |
| *P. elaphoglossoides* Schott | KU739294 | - | KU727507 | - | - |
| *P. erubescens* K. Koch & Augustin | KU739268 | - | KU727514 | KU727554 | DQ870585.1 |
| *P. eximium* Schott | KU739318 | KU739315 | KU727489 | KU727555 | - |
| *P. findens* Croat & Grayum | - | - | - | - | DQ870586.1 |
| *P. fragrantissimum* (Hook.) G. Don | JQ586639.1 | JQ586639.1 | KU727504 | KU727544 | DQ870587.1 |
| *P. glaziovii* Hook. f. | - | KU739352 | KU727605 | KU727632 | DQ870588.1 |
| *P. gloriosum* André | KU739273 | - | KU727498 | KU727564 | DQ870589.1 |
| *P. grandifolium* (Jacq.) Schott | - | - | KU727490 | KU727561 | DQ870590.1 |
| *P. grandipes* K. Krause | - | - | - | - | DQ870591.1 |
| *P. hastatum* K. Koch & Sellow | KU739279 | - | KU727492 | KU727538 | KU727767 |
| *P. hederaceum* (Jacq.) Schott | DQ401355.1 | DQ401355.1 | KU727606 | KU727672 | DQ870613.1 |
| *P. heleniae* Croat | - | - | - | - | DQ870592.1 |
| *P. hopkinsianum* M.L. Soares & Mayo | KU739291 | - | KU727506 | KU727542 | - |
| *P. hylaeae* G.S. Bunting | - | - | - | KU727552 | DQ870593.1 |
| *P. imbe* Schott ex Endl. | KU739299 | - | - | KU727534 | DQ870595.1 |
| *P. inconcinnum* Schott | - | - | KU727584 | KU727633 | - |
| *P. insigne* Schott | KU739286 | - | KU727517 | KU727572 | DQ870596.1 |
| *P. krugii* Engl. | - | - | KU727602 | KU727638 | - |
| *P. lazorii* Croat | - | - | - | - | DQ870597.1 |
| *P. lindenii* Schott | - | - | - | - | DQ870598.1 |
| *P. loefgrenii* Engl. | - | - | - | - | DQ870600.1 |
| *P. longilaminatum* Schott | - | KU739339 | KU727601 | KU727671 | - |
| *P. longistilum* K. Krause | KU739276 | - | KU727491 | - | DQ870601.1 |
| *P. malesevichiae* Croat | - | - | - | - | DQ870602.1 |
| *P. martianum* Engl. | - | KU739311 | KU727488 | KU727559 | - |
| *P. maximum* K. Krause | - | KU739307 | KU727524 | KU727557 | - |
| *P. megalophyllum* Schott | KU739274 | KU739310 | KU727494 | KU727566 | - |
| *P. melanochrysum* Linden & André | KU739323 | KU739341 | - | KU727652 | - |
| *P. melinonii* Brongn. ex Regel | KU739280 | - | - | KU727533 | DQ870603.1 |
| *P. micranthum* Poepp. ex Schott | KU739281 | - | KU727495 | KU727535 | - |
| *P. myrmecophyllum* Engl. | - | - | KU727586 | KU727639 | - |
| *P. nadruzianum* Sakur. | KU739271 | KU739308 | KU727500 | KU727565 | - |
| *P. ornatum* Schott | - | - | - | KU727553 | DQ870604.1 |
| *P. pachyphyllum* K. Krause | - | KU739343 | KU727604 | KU727640 | - |
| *P. panamense* K. Krause | - | - | - | - | DQ870605.1 |
| *P. panduriforme* (Kunth) Kunth | KU739297 | - | KU727525 | KU727573 | DQ870606.1 |
| *P. pedatum* (Hook.) Kunth | KF971326 | KF981852 | KU727501 | KU727530 | DQ870607.1 |
| *P. pinnatifidum* (Willd.) Schott | - | - | - | - | DQ870608.1 |
| *P. pulchrum* G.M. Barroso | KU739275 | - | KU727502 | KU727536 | - |
| *P. quinquelobum* K. Krause | KU739272 | - | KU727529 | KU727578 | - |
| *P. radiatum* Schott | JX024994.1 | JX024994.1 | KU727582 | KU727637 | DQ870610.1 |
| *P. recurvifolium* Schott | - | - | KU727600 | KU727643 | - |
| *P. renauxii* Reitz | KU739278 | - | KU727493 | KU727541 | - |
| *P. rhizomatosum* Sakur. & Mayo | KU739333 | - | - | KU727650 | - |
| *P. roseopetiolatum* Nadruz & Mayo | - | - | - | KU727562 | - |
| *P. ruizii* Schott | - | - | - | - | DQ870611.1 |
| *P. ruthianum* Nadruz | - | KU739345 | - | - | - |
| *P. sagittifolium* Liebm. | - | - | - | - | DQ870612.1 |
| *P. simmondsii* Mayo | KU739320 | KU739340 | KU727581 | KU727636 | - |
| *P. smithii* Engl. | - | - | - | - | DQ870616.1 |
| *P. sphalerum* Schott | KU739269 | - | KU727511 | KU727532 | - |
| *P. squamiferum* Poepp. | KU739288 | KU739344 | KU727516 | KU727548 | DQ870617.1 |
| *P. stenophyllum* K. Krause | - | - | - | - | DQ870618.1 |
| *P. tortum* M.L. Soares & Mayo | - | - | - | KU727556 | - |
| *P. toshibai* M.L. Soares & Mayo | KU739270 | - | KU727496 | KU727563 | - |
| *P. tripartitum* (Jacq.) Schott | KU739321 | KU739348 | KU727603 | KU727635 | DQ870620.1 |
| *P. verrucosum* L. Mathieu ex Schott | - | - | - | - | DQ870621.1 |
| *P. wendlandii* Schott | - | - | - | - | DQ870622.1 |
| *P. wittianum* Engl. | KU739292 | - | KU727505 | KU727550 | - |
| *P. wurdackii* G.S. Bunting | KU739267 | - | KU727522 | - | - |
| *P. linnaei*Kunth, Enum | KU739287 | KU739314 | KU727519 | KU727569 | DQ870599.1 |
| ***P*. subg. *Pteromischum*** |  |  |  |  |  |
| *P. oblongum* (Vell.) Kunth | KF971325 | KF981851 | KU727523 | - | - |
| *P. ochrostemon* Schott | - | - | - | KU727575 | - |
| *P. placidum* Schott | - | - | - | - | DQ870609.1 |
| *P. propinquum* Schott | KU739284 | - | - | KU727574 | - |
| *P. rudgeanum* Schott | - | - | KU727620 | KU727642 | DQ870568.1 |
| *P. schottii* subsp. *talamancae* (Engl.) Grayum | - | - | - | - | DQ870619.1 |
| *P. surinamense* (Miq.) Engl. | - | - | - | KU727628 | - |

**Table 3**. Sequences and references of the primers used to amplify and sequence *matK, trn*K*, trn*L intron*, trn*L-*trn*F intergenic spacer, ETS and 18S.

| **Molecular marker** | **Primer sequence (5’ - 3’)** | **Reference** |
| --- | --- | --- |
| *trn*K *- mat*K | trnK-F: GGGTTGCTAACTCAATGGTAGAG  trnK-R1: GAACCCGGA ACTHGTCGGAT | Wicke and Quandt, 2009 |
| 18S – ETS | ETS-AF: GACCGTGACGGYACGTGAG  18S-R: AGACAAGCATATGACTACTGGCAGG | Gauthier *et al.,* 2008 |
| *trn*L intron | c: 5´ CGAAATCGGTAGACGCTACG 3'  d: 5´ GGGGATAGAGGGACTTGAAC 3' | Taberlet et al., 1991 |
| *trn*L-*trn*F intergenic spacer | e: 5´ GGTTCAAGTCCCTCTATCCC 3'  f: 5´ ATTTGAACTGGTGACACGAG 3' | Taberlet et al., 1991 |

**Table 4.** Liliopsida taxon sampling and accession numbers

| Species | Chloroplast genome accession number |
| --- | --- |
| *Phalaenopsis* *aphrodite* subsp. *formosana* | NC007499 |
| *Dioscorea elephantipes* | NC009601 |
| *Lemna minor* | NC010109 |
| *Typha latifolia* | NC013823 |
| *Phoenix dactylifera* | NC013991 |
| *Spirodela polyrhiza* | NC015891 |
| *Wolffiella lingulata* | NC015894 |
| *Wolffia australiana* | NC015899 |
| *Colocasia esculenta* | NC016753 |
| *Elaeis guineensis* | NC017602 |
| *Phalaenopsis equestris* | NC017609 |
| *Erycina pusilla* | NC018114 |
| *Elodea canadensis* | NC018541 |
| *Heliconia collinsiana* | NC020362 |
| *Zingiber spectabile* | NC020363 |
| *Pseudophoenix vinifera* | NC020364 |
| *Bismarckia nobilis* | NC020366 |
| *Dasypogon bromeliifolius* | NC020367 |
| *Calamus caryotoides* | NC020365 |
| *Cymbidium sinense* | NC021430 |
| *Cymbidium tracyanum* | NC021432 |
| *Cymbidium mannii* | NC021433 |
| *Cymbidium tortisepalum* | NC021431 |
| *Cymbidium aloifolium* | NC021429 |
| *Najas flexilis* | NC021936 |
| *Cocos nucifera* | NC022417 |
| *Musa textilis* | NC022926 |
| *Ravenala madagascariensis* | NC022927 |
| *Curcuma roscoeana* | NC022928 |
| *Fritillaria taipaiensis* | NC023247 |
| *Veratrum patulum* | NC022715 |
| *Dendrobium catenatum* | NC024019 |
| *Dioscorea rotundata* | NC024170 |
| *Cypripedium macranthos* | NC024421 |
| *Calanthe triplicata* | NC024544 |
| *Paris verticillata* | NC024560 |
| *Fritillaria hupehensis* | NC024736 |
| *Fritillaria cirrhosa* | NC024728 |
| *Allium cepa* | NC024813 |
| *Iris gatesii* | NC024936 |
| *Bomarea edulis* | NC025306 |
| *Luzuriaga radicans* | NC025333 |
| *Corallorhiza macrantha* | NC025660 |
| *Corallorhiza trifida* | NC025662 |
| *Corallorhiza odontorhiza* | NC025664 |
| *Corallorhiza wisteriana* | NC025663 |
| *Corallorhiza mertensiana* | NC025661 |
| *Corallorhiza bulbosa* | NC025659 |
| *Ananas comosus* | NC026220 |
| *Calycanthus floridus glaucus* | NC004993 |
| *Drimys granadensis* | NC008456 |
| *Piper cenocladum* | NC008457 |
| *Solanum tuberosum* | NC008096 |
| *Nelumbo lutea* | NC015605 |
| *Brassica napus* | NC016734 |
| *Magnolia officinalis* | NC020316 |
| *Eucalyptus polybractea* | NC022393 |
| *Camellia reticulata* | NC024663 |
| *Macadamia integrifolia* | NC025288 |
| *Oryza sativa* | NC001320 |
| *Zea mays* | NC001666 |
| *Triticum aestivum* | NC002762 |
| *Oryza nivara* | NC005973 |
| *Oryza sativa* | NC008155 |
| *Agrostis stolonifera* | NC008591 |
| *Hordeum vulgare* subsp. *vulgare* | NC008590 |
| *Sorghum bicolor* | NC008602 |
| *Lolium perenne* | NC009950 |
| *Brachypodium distachyon* | NC011032 |
| *Festuca arundinacea* | NC011713 |
| *Dendrocalamus latiflorus* | NC013088 |
| *Anomochloa marantoidea* | NC014062 |
| *Indocalamus longiauritus* | NC015803 |
| *Phyllostachys edulis* | NC015817 |
| *Acidosasa purpurea* | NC015820 |
| *Phyllostachys nigra* var. *henonis* | NC015826 |
| *Bambusa emeiensis* | NC015830 |
| *Ferrocalamus rimosivaginus* | NC015831 |
| *Panicum virgatum* | NC015990 |
| *Leersia tisserantii* | NC016677 |
| *Phyllostachys propinqua* | NC016699 |
| *Rhynchoryza subulata* | NC016718 |
| *Oryza meridionalis* | NC016927 |
| *Oryza rufipogon* | NC017835 |
| *Festuca ovina* | NC019649 |
| *Festuca altissima* | NC019648 |
| *Festuca pratensis* | NC019650 |
| *Lolium multiflorum* | NC019651 |
| *Arundinaria gigantea* | NC020341 |
| *Pharus latifolius* | NC021372 |
| *Triticum monococcum* | NC021760 |
| *Secale cereale* | NC021761 |
| *Triticum urartu* | NC021762 |
| *Aegilops tauschii* | NC022133 |
| *Aegilops speltoides* | NC022135 |
| *Oryza rufipogon* | NC_022668.1 |
| *Setaria italica* | NC022850 |
| *Phragmites australis* | NC022958 |
| *Aegilops geniculata* | NC023097 |
| *Aegilops cylindrica* | NC023096 |
| *Pharus lappulaceus* | NC023245 |
| *Puelia olyriformis* | NC023449 |
| *Deschampsia antarctica* | NC023533 |
| *Sorghum timorense* | NC023800 |
| *Arundinaria appalachiana* | NC023934 |
| *Arundinaria tecta* | NC023935 |
| *Olyra latifolia* | NC024165 |
| *Cenchrus americanus* | NC024171 |
| *Oryza glaberrima* | NC024175 |
| *Digitaria exilis* | NC024176 |
| *Neyraudia reynaudiana* | NC024262 |
| *Lecomtella madagascariensis* | NC024106 |
| *Oryza australiensis* | NC024608 |
| *Bambusa multiplex* | NC024668 |
| *Phyllostachys sulphurea* | NC024669 |
| *Arundinaria fargesii* | NC024712 |
| *Sarocalamus faberi* | NC024713 |
| *Chimonocalamus longiusculus* | NC024714 |
| *Fargesia nitida* | NC024715 |
| *Fargesia spathacea* | NC024716 |
| *Fargesia yunnanensis* | NC024717 |
| *Gaoligongshania megalothyrsa* | NC024718 |
| *Gelidocalamus tessellatus* | NC024719 |
| *Indocalamus wilsonii* | NC024720 |
| *Indosasa sinica* | NC024721 |
| *Oligostachyum shiuyingianum* | NC024722 |
| *Yushania levigata* | NC024725 |
| *Pleioblastus maculatus* | NC024723 |
| *Thamnocalamus spathiflorus* | NC024724 |
| *Ampelocalamus calcareus* | NC024731 |
| *Triticum timopheevii* | NC024764 |
| *Aegilops longissima* | NC024830 |
| *Aegilops bicornis* | NC024831 |
| *Aristida purpurea* | NC025228 |
| *Centotheca lappacea* | NC025229 |
| *Chionochloa macra* | NC025230 |
| *Coleataenia prionitis* | NC025231 |
| *Danthonia californica* | NC025232 |
| *Elytrophorus spicatus* | NC025233 |
| *Eriachne stipacea* | NC025234 |
| *Hakonechloa macra* | NC025235 |
| *Isachne distichophylla* | NC025236 |
| *Monachather paradoxus* | NC025237 |
| *Thysanolaena latifolia* | NC025238 |
| *Echinochloa oryzicola* | NC024643 |
| *Aegilops sharonensis* | NC024816 |
| *Aegilops searsii* | NC024815 |
| *Aegilops kotschyi* | NC024832 |
| *Triticum turgidum* | NC024814 |
| *Triticum macha* | NC025955 |
| *Acorus calamus* | NC007407 |
| *Acorus americanus* | NC010093 |
| *Eustrephus latifolius* | NC025305 |
| *Acorus gramineus* | NC026299 |

**Table 5**. Liliopsida fossils used as calibration points

| Fossil | Age (Ma) |
| --- | --- |
| *Liliacidites* sp. A Doyle & Hickey 1976 | 113.0 – 125.0 |
| *Montrichardia aquatica* F.A.Herrera, C.A.Jaram., Dilcher, S.L.Wing & C.Gómez Nav. 2008 | 58.0 – 60.0 |
| *Thalassites parkavonenses* Benzecry & Brack-Hanes 2008 | 38.0 – 47.8 |
| *Tripylocarpa aestuaria* Gandolfo & Futey 2012 | 63.49 – 64.67 |
| *Mauritiidites crassibaculatus* van Hoeken-Klinkenberg, 1964 [Schrank, 1994] | 66.0 – 72.1 |
| *Sabalites carolinensis* E.W.Berry 1914 | 83.6 – 86.3 |

**Table 6**. Results of approximately unbiased (AU) and Shimodaira–Hasegawa (SH) tests for alternative topological arrangements among *P*. subg. *Philodendron*, *P*. subg. *Meconostigma* and American *Homalomena*.

item obs au np | bp pp kh sh wkh wsh |

1 -31.7 0.998 0.996 | 0.995 1.000 0.987 0.990 0.987 0.990 |

2 31.7 0.005 0.003 | 0.003 2e-14 0.013 0.013 0.013 0.018 |

3 32.9 0.003 0.002 | 0.001 5e-15 0.011 0.011 0.011 0.023 |

Legend:

1. (American *Homalomena* (*P*. subg. *Meconostigma* + *P*. subg. *Philodendron*))

2. (*P*. subg. *Meconostigma* (American *Homalomena* + *P*. subg. *Philodendron*))

3. (*P*. subg. *Philodendron* (*P*. subg. *Meconostigma* + American *Homalomena*))

Figure 1: Gene trees estimated in Mr. Bayes. The posterior probabilities are indicated in the nodes. A. ETS and 18S gene tree; B. *mat*K 5’ portion gene tree; C. *mat*K 3’ portion and *trn*K gene tree; D. *trn*L intron gene tree; E. *trn*L-*trn*F intergenic spacer gene tree.
